# Comparison of Various Indices of Energy Metabolism in Recumbent and Healthy Dairy Cows

**DOI:** 10.1371/journal.pone.0169716

**Published:** 2017-01-20

**Authors:** Hugues Guyot, Johann Detilleux, Pascal Lebreton, Catherine Garnier, Marie Bonvoisin, Frederic Rollin, Charlotte Sandersen

**Affiliations:** 1 Department of Sustainable Animal Production, Fundamental and Applied Research for Animals and Health, University of Liege, Liege, Belgium; 2 NBVC, Early Health Indicators, Dardilly, France; 3 IODOLAB, Marcy l’Etoile, France; 4 Department of Comparative Veterinary Medicine; Fundamental and Applied Research for Animals and Health, University of Liege, Liege, Belgium; Max Delbruck Centrum fur Molekulare Medizin Berlin Buch, GERMANY

## Abstract

**Background:**

Downer cow syndrome (DCS) is often diagnosed in dairy cattle during the early post-partum period. The etiology of this condition is not completely understood, as it can be related to the energetic or electrolyte metabolism, as well as to infectious diseases or to trauma.

**Hypothesis/Objectives:**

The aim of this study is to compare energy metabolism and insulin sensitivity indices and various biochemical parameters between recumbent and healthy dairy cows.

**Animals:**

A prospective study has been undertaken on 361 recumbent and 80 healthy Holstein cows.

**Methods:**

Plasmatic glucose, insulin, non-esterified fatty acid (NEFA) and β-hydroxybutyrate (BHB) were assayed in all cows in order to calculate the insulin sensitivity indices but also minerals (Calcium, Phosphorous and Magnesium), thyroxin and creatine kinase. Body Condition Scores (BCS) was assessed.

**Results:**

Significant differences in NEFA, and the glucose and insulin sensitivity indices (“Homeostasis Model Assessment” HOMA, “Revised Quantitative Insulin Sensitivity Check Index” RQUICKI, RQUICKI-BHB) were observed between healthy and recumbent cows in the early post-parturient period indicating disturbances of glucose and insulin homeostasis in the recumbent cows. In the same manner, mineral concentrations were significantly different between healthy and recumbent cows. Glucose, insulin NEFA, and HOMA, were different between early post-partum downer cows and the DCS-affected cows later in lactation.

**Conclusion and clinical importance:**

Results indicate disturbances in energy homeostasis in DCS-affected dairy cows. Further research should determine a prognostic value of the indices in cows suffering from recumbency of metabolic origin.

## Introduction

Downer cow syndrome (DCS) is defined as lateral or sternal recumbency that persists for longer than 24 hours [1], or that persists for longer than two weeks despite of treatment [2]. The incidence of this syndrome ranges from 4.5 to 14% [3]. Downer cow syndrome can be seen in all stages of the animal’s reproductive cycle but the majority of all downer cows are diagnosed shortly after parturition. A multitude of metabolic, infectious, toxic, degenerative and traumatic disorders may result in recumbency of the animal. The metabolic etiologies of DCS include hyperketonemia and fatty liver syndrome, hypophosphatemia, hypomagnesaemia, and hypocalcemia. A metabolic origin can be suspected from the assessment of the body condition score (BCS) [4] and may further be confirmed by measuring blood metabolic markers. Blood β-hydroxybutyrate (BHB), glycaemia, non-esterified-fatty-acids (NEFA) and blood minerals can be used to evaluate the ration and the energetic metabolism (e.g. fat utilization), which is a major factor contributing to the development of recumbency. Hypothyroidism can be associated to DCS, and measurement of thyroxin (T4) can be used to determine the prognosis of DCS cases.

Most metabolic disturbances result from a dysfunction in glucose metabolism; however, glycemia, insulinemia and NEFA are difficult to be interpreted independently of each other. For measuring insulin sensitivity, different models, varying in complexity and costs, exist. Among these models we can find the “Homeostasis Model Assessment” (HOMA) [5] and their logarithmic or reciprocal score (log HOMA and 1/HOMA) [6], the “Quantitative Insulin Sensitivity Check Index” (QUICKI) [7] and the “Revised Quantitative Sensitivity Check Index” (RQUICKI). RQUICKI had been described by Perseghin and coworkers [8] for humans and has already been applied to healthy cows [9]. RQUICKI-BHB has also been described in Holstein-Friesian cows and has been compared to RQUICKI and glucose tolerance test [10]. Whereas plasma concentrations of glucose, insulin and NEFA vary with the week of lactation, the RQUICKI is not affected by the production period in healthy cows [9].

The aim of this study is to give insulin sensitivity indices and different biochemical metabolites in cows suffering from DCS and to compare these values to those obtained in healthy cows.

## Materials and Methods

### Animals

The protocol was approved by the competent institutional authority for the ethical use of experimental animals (*Commission d’Ethique d’Utilisation des Animaux à l’Université de Liège*). Cows were keptin standard husbandry conditions and were used to being handled by the care taking staff. Primiparous or multiparous dairy cows with the following conditions have been selected: cows in sternal and/or lateral recumbency, unable to get up spontaneously for longer than 24 hours (without prior treatment administered by the owner or veterinarian). Cows with musculoskeletal/neural lesions have been excluded, as well as toxic mastitis, metritis and any other non-metabolic causes of recumbency (based on calving history and complete clinical examination). DCS cows have been attributed to three categories: early post-partum cows (0–8 days) (DCS-early); lactating cows (after 8 days in milk) (DCS-late); and dry cows (DCS-dry). Healthy post-parturient dairy cows (H-early), between 2 to 8 days in milk, have been selected according to the following criteria: normal calving history (no dystocia, no retained fetal membranes, no milk fever, no mastitis), normal complete clinical examination, good appetite (no sub-clinical ketosis, verified by measurement of blood BHB < 1.0 mmol/L, BCS between 2.5 and 3.5 (and no history of fattening during dry period), total protein in serum (TPS) < 81 g/L and difference between TPS and total protein in plasma ≤ 6 g/L (plain and Na-heparin tubes, Vacutainer, Becton Dickinson, VWR, Belgium) were used respectively for total protein in serum and total protein in plasma sampling and measurement was made by electronic refractometer (Electronic refractometer, Euromex, Arnheim, The Netherlands) after centrifugation and separation of serum and plasma). Additional data including breed, age, lactation number, days in milk, BCS and feed composition have also been collected in all groups.

### Samples and analyses

All DCS samples have been collected prior to the administration of a treatment by the veterinarian. Blood was collected from the jugular vein into 3 different tubes (Heparin-Lithium, EDTA and plain tubes, vacutainer, Becton Dickinson, VWR, Belgium). Glycaemia and BHB have been measured immediately using hand-held analyzer (Precision XCeed, Abbott, Wavre, Belgium). Blood analyses, consisting of NEFA, insulin, Ca, P, Mg, CK, T4 have been performed by IODOLAB laboratory in Marcy-l’Etoile, France. NEFA were measured using enzymatic colorimetry endpoint kit (Randox Laboratories, Crumlin, United Kingdom). Creatine kinase activity has been measured by UV kinetic colorimetry (Hitachi 717 automate, DiaSys, Condom, France), triggering substrate at 37°C; total Ca and P were assayed with UV colorimetry endpoint (respectively phosphonaso III and phosphomolybdate); and Mg was tested by Xylidyle blue colorimetry (Magnesium Colorimetric Assay Kit, Adipogen, Liesthal, Switzerland). T4 and insulin have been measured by Radio-Immuno-Assay (DiaSorin, Dietzenbach, Germany). Normal ranges for the NEFA and BHB were determined based on the literature [11]. For all other assays, the normal ranges were determined by NBVC’s laboratory, based on their own studies in healthy cows.

### Calculated indices

The HOMA index was determined by the formula “Glucose (mmol/L) x Insulin (μU/ml)” [5]. The QUICKI was calculated as follow: “1 / [log (glucose) + log (insulin)]” [7]. The RQUICKI was calculated with the formula of Perseghin and coworkers [8]: RQUICKI = 1 / [log (glucose) + log (insulin) + log (NEFA)]. The RQUICKI-BHB was calculated according to the formula of Balogh and coworkers [10]: 1 / [log (glucose) + log (insulin) + log (NEFA) + log (BHB)]. For the “QUICKIs”, glucose is expressed in mg/dl, insulin in μU/ml, NEFA in mmol/L and BHB in mmol/L.

### Statistical analysis

The following descriptive statistics of the group populations DCS-early, DCS-late, DCS-dry, and H-early have been realized: population size, distribution of ranks of lactation, mean ± standard deviation (SD) and median with percentiles 2.5 and 97.5 for BCS, age and the different bloods parameters. To compare the different populations, test values were transformed (Boc Cox transformation, proc Transreg, SAS) to approach normal distributions: logarithmic transformation for age and glucose, root power (0.25) for CK and T4, root power (0.5) for rank of lactation, NEFA, insulin and minerals. Insulin sensitivity index (HOMA, QUICKI, RQUICKI, RQUICKI-BHB) and other values (glucose, NEFA, Insulin, BHB, Ca, P, Mg, CK, T4) were compared between populations DCS-early and H-early, DCS-early and DCS-late, and DCS-early and DCS-dry, in a one-way analysis of variance (proc GLM, SAS). Differences have been considered significant at values of p < 0.05.

## Results

### Population characteristics

Data were obtained from 361 recumbent Holstein Frisian cows, originating from 302 different herds in France and Belgium (1 to 4 animals per farm) and 80 healthy Holstein Frisian cows from 16 different farms where no DCS was recorded (5 animals per farm). Samples were always taken during winter season over a period of three consecutive years. Details of the different groups are presented in Table 1. The mean number of lactations in DCS cows was significantly higher in the DCS-dry group (5 ± 3) than in DCS cows in the postpartum period and in lactation (both 4 ± 2, p < 0.001). The mean BCS score of DCS-early cows was 3.4 ± 0.7 and was significantly higher (p < 0.001) than in the DCS-late group (2.6 ± 0.3) but also significantly higher (p<0.0001) than in healthy cows (2.8 ± 0.3).

**Table 1:** Main characteristics of the different populations studied.

	Healthy-early lactation	DCS-early lactation	DCS-late lactation	DCS-dry period
Number of animals	80	263	69	29
Multiparous	77 (96%)	249 (95%)	61 (88%)	28 (97%)
Primiparous	3 (4%)	14 (5%)	8 (12%)	1 (3%)
Parity	4 ± 2	4 ± 2	4 ± 2	5 ± 3^a^
	2-3-7	1-4-8	1-3-9	2-4-11
BCS	2.8 ± 0.3	3.4 ± 0.7^b^	2.6 ± 0.8^c^	3.0 ± 0.8
	2.5–3.0–3.5	2.0–3.5–4.5	1.2–2.5–4.0	2.0–3.0–4.5

Data represented as mean ± standard deviation and as 2.5–50–97.5 percentiles.

DCS = downer cow syndrome.

^a^ = significantly different from DCS cow in lactation (p<0.001);

^b^ = significantly different from healthy cows (p<0.001)

^c^ = significantly different from DCS cows in the pearly lactation period (p<0.001).

Basic composition of the diet in the farms enrolled in the study revealed that 55% gave a mixed ration with grass and maize silage, 25% with grass (silage), 15% maize and beet pulp silage and 5% others (dry rations). Minerals and concentrates were present in all of the rations which had been standardized according to the recommendations of the French Institute of Agricultural Research [12]. The type of the ration had no effect on any of the blood parameters (p>0.1).

### Insulin sensitivity index, Glucose, Insulin, NEFA, BHB

The different parameters of insulin sensitivity are presented in Table 2. Hyperglycemia (> 75 mg/dL) was observed on 44% of DCS cows and hypoglycemia (< 45 mg/dL) in 15% of them. The mean blood glucose concentration of all DCS together was 80 ± 50 mg/dL. Each group individually had significantly higher mean blood glucose concentrations than the group of healthy cows, which had a mean blood glucose concentration of 48 ± 8 mg/dL. However, mean blood glucose concentration was lower in post-parturient than in lactating cows with DCS.

**Table 2:** Glucose, NEFA, Insulin, BHB concentrations; HOMA, QUICKI, RQUICKI and RQUICKI-BHB indices in the different populations.

	Healthy-early lactation	DCS-early lactation	DCS-late lactation	DCS-dry period
Glucose (mg/dL)	48 ± 8	78 ± 42^a^	92 ± 78^b^	76 ± 34^a^
	37-47-64	21-68-157	23-79-194	35-65-145
NEFA (mmol/L)	0.45 ± 0.25	0.98 ± 0.39^a^	0.75 ± 0.44^b^	0.93 ± 0.33^a^
	0.08–0.41–0.97	0.34–0.90–1.86	0.20–0.70–1.64	0.42–0.83–1.64
Insulin (μU/ml)	17 ± 6	16 ± 9	18 ± 10^b^	18 ± 9
	7-18-26	4-14-38	4-16-38	3-18-34
BHB (mmol/L)	0.55 ± 0.21	0.79 ± 0.91	0.85 ± 1.32	1.07 ± 1.37
	0.20–0.60–0.93	0.10–0.52–3.88	0.01–0.40–5.00	0.10–0.70–3.91
HOMA	47 ± 19	69 ± 57^a^	92 ± 85^b^	83 ± 69^a^
	18-43-88	8-58-198	15-72-311	7-62-259
QUICKI	0.35 ± 0.03	0.34 ± 0.05	0.33 ± 0.04	0.34 ± 0.05
	0.31–0.35–0.40	0.28–0.33–0.46	0.27–0.32–0.41	0.27–0.33–0.48
RQUICKI	0.41 ± 0.06	0.34 ± 0.06^a^	0.35 ± 0.05	0.34 ± 0.06^a^
	0.34–0.41–0.56	0.27–0.34–0.50	0.27–0.35–0.47	0.27–0.33–0.51
RQUICKI-BHB	0.47 ± 0.08	0.39 ± 0.10^a^	0.42 ± 0.12	0.39 ± 0.13^a^
	0.38–0.46–0.62	0.27–0.38–0.60	0.25–0.41–0.70	0.25–0.34–0.64

Data represented as mean ± standard deviation and as 2.5–50–97.5 percentiles.

DCS = downer cow syndrome. NEFA: Non-Esterified Fatty Acids. BHB: Beta-hydroxy-butyrate. HOMA: Homeostasis Model Assessment. QUICKI: Quantitative Insulin Sensitivity Check Index. RQUICKI: Revised Quantitative Insulin Sensitivity Check Index.

^a^ = significantly different from healthy cows (p<0.001);

^b^ = significantly different from DCS cows in the early lactation period (p<0.001).

Mean NEFA concentrations of DCS-cows were above the normal limits determined by Van Winden and coworkers [11], who set upper limits of <0.7 mmol/L in lactation and <0.4 mmol/L at the end of gestation. Mean NEFA concentration of DCS-early was significantly (p < 0.0001) higher than group H-pp and significant higher (p < 0.0001) than in group DCS-late.

Insulinemia in DCS groups was within the normal range (NBVC normal range: 10–50 μU/L). There was no significant difference between healthy and DCS cows in the postpartum period (p > 0.1) but the mean blood insulin level was significantly higher group DCS-late (p < 0.05).

Mean BHB concentrations of all groups affected by DCS were within the normal range of < 1.2 mmol/L [11]. No significant difference in mean values for BHB between the populations DCS-early and H-early existed. The QUICKI index was not different among groups (p > 0.1).

The HOMA index was significant different (p < 0.05) between H-early and DCS-early cows and between H-early and DCS-late cows. For RQUICKI, healthy postparturient cows showed higher values compared to all groups of DCS cows (p < 0.0001). TRQUICKI-BHB had the same statistical trend as RQUICKI.

### Relationship between BCS and RQUICKI

For all DCS-affected cows, the average of RQUICKI for cows with BCS < 3 was 0.37 ± 0.05. This represented a significant difference (p < 0.05) with the RQUICKI of cows with BCS ≥ 3 who had a mean RQUICKI of 0.34 ± 0.05. No significant difference (p > 0.05) has been revealed for RQUICKI of cows with a BCS of 3–3.5 and cows with a BCS ≥ 4.

### Minerals (Ca, P, Mg) & other blood parameters (CK and T4)

Details of calcium, phosphorus, magnesium, creatine kinase and thyroxine blood concentrations in the different populations are displayed in Table 3. Only mean calcium concentration of the groups DCS-dry and H-early were within the normal range of 2.17–2.85 mmol/L. Mean phosphorous concentration was below normal range in the group of postpartum DCS cows (normal range: 1.45–2.58 mmol/L). The mean calcium and phosphorus concentrations of the group DCS-early were significantly lower than those of group H-early and DCS-late (p < 0.001). In the same way, the group DCS-dry had significantly higher concentrations for Ca and P compared to DCS cows in lactation (p < 0.05). The mean magnesium concentration of all populations was within the normal range (normal range: 0.76–1.43 mmol/L) but the group DCS-early had significantly higher concentrations than healthy and lactating DCS cows (p < 0.05).

**Table 3:** Plasmatic Calcium, Phosphorous, Magnesium, CK and T4 concentrations in the different populations.

	Healthy-early lactation	DCS-early lactation	DCS—late lactation	DCS—dry period
Calcium (mmol/L)	2.24 ± 0.21	1.57 ± 0.76^a^	2.06 ± 0.56^b^	2.19 ± 0.83^c^
	1.72–2.25–2.52	0.50–1.54–2.92	0.90–2.09–2.73	0.87–2.22–4.21
Phosphorus (mmol/L)	1.74 ± 0.32	1.21 ± 0.89^a^	1.67 ± 0.93^b^	1.97 ± 1.01^c^
	1.04–1.77–2.29	0.17–1.03–3.51	0.21–1.66–3.18	0.35–1.80–4.10
Magnesium (mmol/L)	0.87 ± 0.12	1.16 ± 0.55^a^	1.00 ± 0.52^b^	1.06 ± 0.47
	0.62–0.89–1.08	0.30–1.10–2.22	0.16–1.00–1.86	0.23–1.04–1.90
CK (UI/L)	120 ± 50	1466 ± 2069^a^	1446 ± 1209	1807 ± 1269
	62-106-241	82-772-4672	105-1102-3863	60-2141-3000
T4 (nmol/L)	34 ± 11	27 ± 13^a^	35 ± 13^b^	22 ± 14^c^
	17-33-58	7-24-58	6-31-80	7-17-52

Data represented as mean ± standard deviation and as 2.5–50–97.5 percentiles.

DCS–downer cow syndrome; CK = creatine kinase; T4 = thyroxine;

^a^ = significantly different from healthy cows;

^b^ = significantly different from DCS cows in *the early lactation* period;

^c^ = significantly different from DCS cows in early and late lactation.

Thyroxin levels were significantly in DCS-early cows than in lower than healthy and lactating DCS cows (p < 0.05). The mean T4 concentration of cows in group DCS-dry was significantly lower than in lactating DCS cows (p < 0.01). The mean T4 concentration of healthy cows was at the lower end of the normal range for dairy cattle [13] while for DCS cows the T4 concentrations may indicate a hypothyroid condition. Mean CK values were higher than the reference range of the laboratory (< 1236 UI/L). Mean CK concentration was higher in group DCS-early than in group H-early (p < 0.0001).

## Discussion

The central aim of this study was to determine the insulin sensitivity indices in cows affected by DCS. Mean values of blood glucose, insulin, NEFA and BHB, as well as the calculated indices of HOMA, QUICKI, RQUICKI and RQUICKI-BHB in healthy peri-parturient cows as well as in DCS-affected cows in different periods of their productive cycle are reported. Glucose, NEFA, HOMA, RQUICKI and RQUICKI-BHB are different between healthy and DCS cows in the first 8 days after calving. Mean values of glucose, NEFA, insulin, and HOMA are different between DCS affected cows in the first eight days of calving compared to later periods of lactation. There was no significant difference among any of the groups for HOMA and BHB.

The results for RQUICKI and RQUICKI-BHB seem particularly interesting as they allow differentiation of healthy cows from those affected by DCS in the early period of the production cycle. Cows that suffer from DCS in a period later than 8 days from calving have RQUICKI and RQUICKI-BHB values which are similar to those obtained in healthy post-parturient cows. This indicates that DCS in the direct post-parturient period is related to profound metabolic disturbances, while later DCS may not. Holtenius and Holtenius [9] reported the RQUICKI of healthy dairy cows to be of 0.48 ± 0.15, which is comparable with the values obtained in healthy cows in the present study. They further report that values are not varying during the first weeks of lactation. Reports on metabolic indices in sick cattle are relatively sparse but use of RQUICKI [14] and RQUICKI-BHB [10] has already been described in dairy cows suffering from ketotic conditions.

The higher BCS in the DCS-early cows may explain the difference in insulin sensitivity. Insulin resistance can be described as a decrease in responses or sensitivity to the metabolic actions of insulin like, e.g. the inhibition of hepatic glucose synthesis. At the beginning of lactation, insulin resistance with low insulin concentrations occurs in dairy cows allowing them to diverge the glucose catabolism in insulin-responsive tissues to lactose production in the mammary gland. As the milk production increases, in parallel to a negative energy balance, fat mobilization compensates the energy deficits [15,16]. Consequently, the insulin resistance can reduce the responses of adipose tissues to inhibitory effects of insulin. This may increase lipid mobilization and provoke fatty liver and ketosis [17]. In dairy cows, numerous inflammatory diseases (metritis, mastitis, orthopedic diseases, subacute ruminal acidosis) can affect insulin sensitivity and potentially promote lipolysis, ketogenesis and hepatic diseases [18, 19, 20]. Fat cows with high BCS, may also have their insulin sensitivity reduced [9].

But other factors than high BCS and obesity may determine insulin sensitivity. Several researchers found a clear inverse relationship between insulin sensitivity assessed by RQUICKI and BCS [21, 22] while others did not [14]. In our study, DCS cows with low BCS (< 3) have significantly higher insulin sensitivity than cows with BCS ≥ 3. However, no relationship between RQUICKI and BCS has been detected. Moreover, the evolution of BCS, and especially important weight loss after calving, is a better parameter to estimate the insulin sensitivity rather than a punctual measure of BCS. The relationship between RQUICKI and BCS in healthy cows has not been measured, as BCS of 2.5 to 3.5 was a selection criterion and would have caused selection bias. The mean BCS of group DCS-early is higher than that of group H-early which underlines that cows with higher BCS are more susceptible to develop DCS in the postpartum period. It is generally accepted that ruminants with excessive BCS ante-partum (BCS ≥ 4) have a greater risk of metabolic problems because of excessive mobilization of body reserves [17]. Among the metabolic etiologies of DCS, the fatty liver syndrome appears to be the most commonly observed condition in the first month after calving in cows with BCS > 3.5 [22]. In the present study, the blood BHB concentration was within the normal range in DCS cows. BHB must be interpreted in correlation with BCS because if BCS is low, there are no further fat stores for mobilization and transformation into NEFA or ketone bodies.

Other studies found RQUICKI able to differentiate healthy herds from herds with a high incidence of displaced abomasum [23]. In the present study, most of the DCS cows also showed hyperglycemia and high NEFA concentrations. An increased plasma NEFA level results from adipose tissue mobilization during the negative energy balance in early lactation [24, 25]. Rising plasma NEFA levels contribute to insulin resistance by suppression of glucose uptake in adipose tissues and muscles [26]. The composition of the diet is of particular importance during the dry period because it modifies the insulin sensitivity, the plasma NEFA concentration and the glycemia [17]. Hyperglycemia can be due to a metabolic stress or recumbent position and increases the risk of left displaced abomasum [27].

As in the present study animals were sampled before the first treatment, the degree of hypocalcaemia and hypophosphatemia in DCS-early cows was pronounced. This is in contrast to most field trials where animals are sampled after the first treatment and thus the potential differences to healthy cows can be biased. The hypocalcemia observed in lactating DCS cows in our study can suggest the potential presence of milk fever. It should be noticed that hypophosphatemia is often associated with hypocalcemia because during hypocalcemia, more phosphate is excreted increasingly in saliva and urine [28]. Hypermagnesemia could have been expected as milk fever increases the secretion of parathormone, involving Mg and Ca re-absorption and thus higher Mg concentrations. In our study, magnesium values were within normal range.

BHB concentrations were within normal range for the large majority of DCS cows, indicating that DCS cows did not show subclinical or clinical ketosis. This suggests that ketosis was not the most common cause of recumbency in our study. However, other authors report subclinical ketosis (> 1.2 mmol/L) in the first week of lactation in almost 25% of cows [29].

CK levels are known to increase 18 to 24 hours after the start of recumbency [30]. As expected in our study, mean CK activity was quite high and 20% above upper the normal range in DCS cows. This suggests that animals were recumbent for an extended period before veterinary advice was sought. Shpigel and coworkers [31] showed that high CK concentrations are associated with a poor prognosis in recumbent cows.

The low T4 concentration in the group of DCS-early can be explain by the observation that plasma T4 concentration progressively increases during pregnancy and thereafter decreases by 50% at calving to re-increase again progressively during lactation. Low T4 hormone concentration at calving may persist during postpartum period when high milk production is associated with negative energy balance [32]. Numerous other causes can explain these low T4 concentrations, such as “stress” [33], metabolic disorders and acute [34] or chronic [35] inflammation which can lead to the Euthyroïd Sick Syndrom [36]. These conditions cannot be excluded in the DCS cows in the present study.

Compared to humans, where RQUICKI is assessed during fasting conditions, conditions may be slightly different in cows. Lactating dairy cows present a different metabolic status (hypoinsulinemia, hypoglycemia) compared to diabetic human patients (hyperinsulinemia, hyper-glycaemia). Caution must also be taken during sample taking as the animal should not be stressed (resulting in hyperglycemia, increased NEFA/insulin) and the moment of the day may also modify these metabolite concentrations. Handling, delay before analysis and the laboratory itself are of considerable importance and may affect the results. This makes the comparison between different studies relatively difficult. The breed of cattle may also influence these indices [37].

Unfortunately, the study has not been designed prospectively. It would have been desirable to include a larger number of animals in the DCS-early group, which would have allowed us to separate between hypocalcemic and non-hypocalcemic cows. Further, it was not possible to include a higher number of primiparous cows. Healthy cows have been chosen deliberately form different farms. These farms have been selected because they had a very low incidence of DCS. Like this we thought to reduce the potential bias from subclinical metabolic disease. However, direct comparison of DCS and healthy cows, exposed to the same nutritional and husbandry environment would have been of interest.

## Conclusion

The present study provides a range of values of insulin sensitivity indices in healthy dairy cattle and in cattle suffering from DCS. The indices HOMA, RQUICKI and RQUICKI-BHB indices presented significant differences allowing discrimination between healthy and DCS cattle. RQUICKI-BHB does not add further information compared to RQUICKI. Lower RQUICKI and higher HOMA are indicative of decreased insulin sensitivity in DCS cows. RQUICKI is routinely used to assess insulin sensitivity in metabolic trials in cattle [20, 23, 38] and it helps us to better define the metabolic disturbances. Further, research should compare RQUICKI in various defined groups of metabolic diseases, such as ketosis type I and II, milk fever in early stages, and pre-partum negative energy balance underline their utility in routine cattle practice beyond research models. Finally, the prognostic value of these indices should be assessed.

## Abbreviations

BCS: body condition score
BHB: β-hydroxy butyrate
CK: creatine kinase
DCS: downer cow syndrome
DCS-dry: downer cow syndrome during post dry period
DCS-early: downer cow syndrome during post parturient period
DCS-late: downer cow syndrome during lactation
H-early: healthy cows during post parturient period
HOMA: homeostasis model Assessment
NEFA: non-esterified fatty acid
QUICKI: quantitative insulin sensitivity check index
RQUICKI: revised quantitative insulin sensitivity check index
T4: thyroxine
TPS: total protein in serum
